# A probabilistic framework for effective battery energy storage sizing in microgrids with demand response

**DOI:** 10.1038/s41598-026-35145-w

**Published:** 2026-03-13

**Authors:** Nehmedo Alamir, Salah Kamel, Tamer F. Megahed, Maiya Hori, Sobhy M. Abdelkader

**Affiliations:** 1 Department of Electrical Engineering, Faculty of Engineering, Aswan University, 81542 Aswan, Egypt; 2 Electrical Power Engineering, Egypt-Japan University of Science and Technology, New Borg El-Arab City, 21934 Egypt; 3 Department of Electrical Engineering, Faculty of Engineering, Mansura University, 35516 Mansoura, Egypt; 4 Tottori University of Environmental Studies, 1-1-1 Wakabadai-kita, Tottori, 689-1111 Japan

**Keywords:** Point estimation method, Battery energy storage, Sizing, Equilibrium optimizer, Microgrid, Energy management, Uncertainty, Batteries, Electrical and electronic engineering

## Abstract

Microgrids (MGs) are increasingly integrating Battery Energy Storage Systems (BESSs) to improve operational flexibility and minimize overall costs. However, probabilistic BESS sizing remains computationally demanding due to uncertainties associated with renewable energy generation, load demand, and market price volatility. This paper presents a hybrid probabilistic sizing framework that integrates the 2m + 1 Point Estimation Method (PEM) with the Equilibrium Optimizer (EO), referred to as the EO–PEM approach. Unlike conventional Monte Carlo simulation–based formulations, the presented method embeds EO within the PEM uncertainty evaluation loop, enabling accurate results with substantially reduced computational effort. Additionally, an incentive-based Demand Response (IDR) model is integrated into the Energy Management (EM) framework. The main objective of the EM is to minimize operational costs and maximize the MG operator’s benefits while ensuring customer satisfaction. Simulation results from the test MG system confirm the superiority of the EO over other applied optimization techniques in solving the deterministic EM problem without BESS. Under uncertainties, the EO–PEM method identifies an optimal BESS capacity of 1 kWh, achieving a reduction in the expected operational cost while maintaining high computational efficiency and robustness. Overall, the results demonstrate the effectiveness of the EO–PEM framework for probabilistic BESS sizing under multi-source uncertainties.

## Introduction

Nowadays, main power grids are undergoing significant transformations due to the increasing integration of distributed microgrids (MGs). MGs, with their high flexibility and operational autonomy, enhance economic performance, improve system reliability, and mitigate environmental impacts.Additionally, MGs provide a regulated framework for integrating distributed energy resources (DERs)^1^. Renewable energy resources (RESs), particularly solar and wind energy, are among the most effective and efficient solutions to the pressing issues of fossil fuel depletion, carbon emissions, and rising energy demand^2^.

The primary goal of incorporating BESS into MGs is to generate optimal power while minimizing the operating costs. Consequently, integrating BESS with MGs in the presence of RES, despite its intermittent nature, and Distributed generators (DGs) reduces system uncertainty, enhances system reliability, and provides economic benefits when BESS is operated optimally through coordinated charging/discharging of the power to MG^3^.

The main objectives of MG resources scheduling are minimization of the operating cost and reducing the power exchanged with the main grid. In literature, most work focuses on the deterministic EM for cost minimization of MG without considering the uncertainty of the generation from RESs such as photovoltaic (PV), wind turbine (WT), total load demand, and energy prices. In^4^, a multi-layer multi- objective technique is proposed to minimize the operating cost, maximization of the benefit through the demand response program (DRP), minimization of greenhouse gas emissions, and enhancement of Multi-Microgrid (MMG) reliability. The multi-objective PSO used in^5^ to solve the EM considering the generation from RESs and market prices. While Ref.^6^, the EM cost minimization problem is solved in MMG based on distributed algorithms by Alternating Direction Method of Multipliers (ADMM) to reduce the emissions while maintaining the operational constraints. In^7^, a Mimosa pudica-based EM has been designed for optimal scheduling of MG. Alamir et al.^8^ use the Honey Badger algorithm for optimal operation and cost minimization while maximizing of the MG operator (MGO) benefit. Alamir et al.^9^ proposed a hybrid technique for minimizing the operation cost with considering the demand response based on Pelican Optimization Algorithm. In addition, a Modified Student Psychology-Based Optimization (MSPBO) is proposed in^10^ for optimizing the operation of MMG resources. A Spotted hyena optimizer (SHO) is implemented to minimize costs and allocate capacitors in MG^11^. However, these researchers did not consider the proper integration of BESS.

Different strategies have been employed in the literature to determine the optimal EM for MG and MMG in the presence of BESS by minimizing the operating cost of MG^12–19^ or enhancing the reliability in addition to cost minimization^20–22^. In^12^, the problem of BESS sizing in MG is solved by using mixed-integer linear programming (MILP) with considering the technology of depth of discharge (DOD) and replacement year. A genetic algorithm (GA) for BESS sizing is proposed in^13^, and the fuzzy system is used to set the power output of the BESS. An improved bat algorithm (IBA) is proposed in^14^ for the optimal operation of MG with BESS. Ref.^15^ used the Simulated Annealing (SA) algorithm to optimize the battery size in PV/wind integrated energy. An Improved Arithmetic Optimization Algorithm (IAOA) is proposed in^16^ for the optimal sizing of MG resources and BESS to reduce the net present cost of the MG system. Water Cycle Algorithm (ACA) is used in^17^ for the optimal operation of MG while minimizing the operating cost; however, the sizing of the BESS was not considered. The sizing problem is solved by grey wolf optimization (GWO)^18^ for cost minimization. In^20^, Mixed-Integer Second Order Cone Programming (MISOCP) is utilized to enhance the reliability of an island MG. However, the optimal sizing for BESS was not considered. MILP with the ε-constraint method is proposed in^21^ for reliability enhancement by minimizing the loss of load expectation. MILP is proposed in^22^ for the optimal BESS sizing for minimization of the investment cost of the BESS, as well as expected MG operating cost, while satisfying the reliability criterion. In^23^, a MILP is proposed for optimizing operating costs for the grid-connected and the island modes of MG considering initial investments cost and the lifetime of BESS.

The aforementioned studies solve the EM problem deterministically without considering the uncertainty of generating patterns, load demand, and market prices. Deterministic strategies rely on the precision of the input data, whereas there are inaccuracies in the input data prediction for EM of MGs. In an accessible power market, market prices and load demand are more uncertain than before^24^. Furthermore, wind and solar generation units fluctuated due to the random behavior of wind speed and solar irradiation^4^. Therefore, a new circumstance should be considered to re-evaluate the effectiveness of the traditional optimization methods. In this regard, new methods must be utilized to account for the intermittent nature of random input data and to limit the risk associated with the design and EM of MGs operating under uncertainty.

The probabilistic methods for solving the EM problem, which consider the uncertainty in input random values, can be classified into three categories: Monte Carlo simulation (MCS), Analytical approaches, and approximation approaches^25^. MCS is employed to determine the optimal sizing problem for the optimal sizing^26–29^. A stochastic approach for the optimal sizing of BESS for cost reduction in MG is presented in^26^. The battery bank is used to supply the load during the peak period and charge during off-peak periods. The uncertainty in load demand and initial State of Charge (SoC) were treated using a MCS.  A probabilistic method for the optimal sizing of ESS, considering the uncertainty in load demand is discussed in^27^. For optimal sizing of BESS, the authors consider the real-time thermal rating in a distribution network. A probabilistic method is proposed in^28^ for the optimal sizing of BESSs with time-of-use (ToU) pricing is implemented for demand response(DR) management. The proposed method considers the uncertainties that inevitably influence the estimate of the customer’s overall cost, such as load demand, energy prices, and economic factors. GA with MCS is employed to solve the uncertainty in generation and consumption for BESS sizing in^29^. Other literature used analytical approaches for optimal sizing^30–32^. In^30^, a sharing-based energy storage system architecture was proposed. The optimal storage system size was found using an analytical method based on a stochastic customer demand model. In^31^, an analytical technique with MCS for the optimal sizing of battery energy storage systems minimizes the total cost paid by the MG’s owner. The proposed technique considered the uncertainty in energy cost, load demand, renewable generation, and discount rate. Ref.^32^, an analytical probabilistic BESS’s sizing approach takes into account the uncertainties of energy price, load demand, power generation, and the parameter that affects the battery lifetime.

Although the existing literature includes different strategies that take into account the impact of uncertainty on sizing, they either used a MCS or analytical methods. For solving the problem MCS in each simulation, employs a deterministic algorithm; its primary limitation is that, to attain the convergence a large number of runs are needed. In the analytical methods, a certain mathematical assumptions simplify the task of studying the statistical characteristics of a random output variable^33^, therefore, the optimal or accurate results cannot be produced^34^. None of the existing literature uses approximations methods for the sizing process of the BESS with consideration of the uncertainty. Point Estimation Method (PEM) is one of the approximations methodologies; PEM has been proven to give an accurate result compared with other methods for uncertainty consideration^35^. PEM uses a deterministic strategy to address probabilistic problems, similar to MCS, but with fewer simulations.

For solving solve the EM problem, PEM are used to consider the uncertainties that exist in MG resources. 2m PEM was proposed for uncertainty consideration in^36–38^. In Ref.^36^, a probabilistic modelling based on the 2m PEM approach is adopted, taking into account Wind and solar power uncertainty as well as market bid variance. The EM problem in^37^ is solved using 2m PEM with a gravitational search algorithm (GSA) with existing uncertainties. Genetic Algorithm (GA) with 2m PEM were employed in^38^ to reduce the total cost of MG. The uncertainty of wind generation and the load demand were considered.

Different optimization techniques with 2m + 1 PEM were used for uncertainty consideration^25, 39, 40^. For reducing the operating cost and improving the reliability^39^, employed PSO technique. ^25^, proposed an EM in MG to reduce operating costs and power transactions with the main grid and maximize the MGO benefit. The proposed EM takes into account the uncertainty in renewable sources generation (PV and wind), energy cost, and load demand using Artificial Hummingbird Algorithm (AHA). An adaptive modified firefly optimizations technique was utilized in^40^. However, the previous studies did not involve the BESS optimal sizing.

Demand response can be defined as a tariff that incentivizes the customers to alter their electricity consumption in accordance with the electricity price change or in the case of grid reliability issues^41^. The two types of DR are price-based DR (PDR) and incentive-based DR (IDR). In the first type, the electricity price varies over the periods of the day, while in the latter, the customers receive the incentive for their reduction or change in consumption^42^. A summary of the relative literature regarding different terms is shown in Table 1.

**Table 1:** Related literature summary.

Ref.	The Objective	Formulation	Uncertainty Modelling	(DR)	BESS	BESS sizing technique
^5^	Cost min.	PSO	✘	✘	✘	-
^6^	Cost and emission min.	ADMM	✘	**✓**	✘	-
^7^	cost min.	Mimosa pudica	✘	✘	✘	-
^8^	Cost min. & benefit max.	HBA	✘	**✓**	✘	-
^9^	Cost min & MGO benefit max.	POA	✘	**✓**	✘	-
^10^	Cost min., benefit max. & peak load reduction	MSPBO	✘	**✓**	✘	-
^11^	Cost min.	SHO	✘	✘	✘	
^12^	Cost min	MILP	✘	✘	**✓**	deterministic
^13^	Cost min	GA	✘	✘	**✓**	deterministic
^14^	Cost min.	IBA	✘	✘	**✓**	deterministic
^15^	Cost min.	SA	✘	✘	**✓**	deterministic
^16^	Net present cost min	IAOA	✘	✘	**✓**	deterministic
^17^	Cost min.	WCA	✘	✘	**✓**	-
^18^	Cost min.	GWO	✘	✘	**✓**	deterministic
^20^	Cost min. &reliability	MISOCP	**✓**	✘	**✓**	-
^21^	Cost min. &reliability	MILP	✘	**✓**	**✓**	deterministic
^22, 23^	Cost min. &reliability	MILP	✘	**✓**	**✓**	deterministic
^26^	Cost min. & battery lifetime	Rule-based control	**✓**	✘	**✓**	MCS
^27^	Peak load shaving	-	**✓**	✘	**✓**	MCS
^28^	reduce the electricity bill	-	**✓**	**✓**	**✓**	MCS
^29^	maximizing the system’s Self Sufficiency Ratio and Net Present Value	GA	**✓**	✘	**✓**	MCS
^30^	Cost min.	-	**✓**	✘	**✓**	analytical method
^31^	Cost Min.	-	**✓**	✘	**✓**	analytical method &MCS
^32^	User benefit max.	-	**✓**	**✓**	**✓**	analytical method
Proposed	Cost Min & MGO benefit max.	EO	**✓**	**✓**	**✓**	PEM

### Contribution

This paper presents an effective probabilistic sizing approach for BESS in energy management for MG. This approach utilizes PEM to reduce the computational efforts needed for probabilistic determination. The effectiveness of presented probabilistic sizing method for BESS in the MG is validated using an advanced optimization algorithm called Equilibrium Optimizer (EO)^43^. The main contributions of this paper are summarized as follows:

- Applying the Equilibrium Optimizer (EO) to microgrid energy management for solving both deterministic and stochastic energy management problems with integrated demand response.
- Developing an EO–PEM hybrid probabilistic sizing framework in which the Equilibrium Optimizer is embedded within the Point Estimation Method uncertainty loops, enabling accurate probabilistic analysis with a significantly reduced computational burden compared to conventional BESS sizing techniques.
- Presenting a computationally efficient probabilistic BESS sizing approach that determines the optimal BESS capacity while accounting for uncertainties in photovoltaic generation, wind power, load demand, and electricity price signals, achieving accuracy comparable to full Monte Carlo simulation at a fraction of the computation time.
- Incorporating an incentive-based demand response model based on customer benefit functions to evaluate its impact on optimal BESS sizing and to provide insights into operator–customer interactions within microgrids.

### Paper organization

This paper’s remaining sections are organized as follows: the mathematical Model for MG component is presented in the section "Mathematical modeling and system configuration". The formulation of EM problem is discussed in the section "Formulation of the energy management problem". The section "Probabilistic operation and uncertainty modeling of MG" presents the probabilistic operation and uncertainty modeling of MG. The section "The probabilistic battery energy storage sizing approach" presents the probabilistic sizing approach for BESS. The formulation of the EO algorithm used for solving the EM are described in the section "Solution method". The simulation results are presented in the section "Simulation results". Finally, the paper is concluded in the section "Conclusions".

## Mathematical modeling and system configuration

**Fig. 1:**
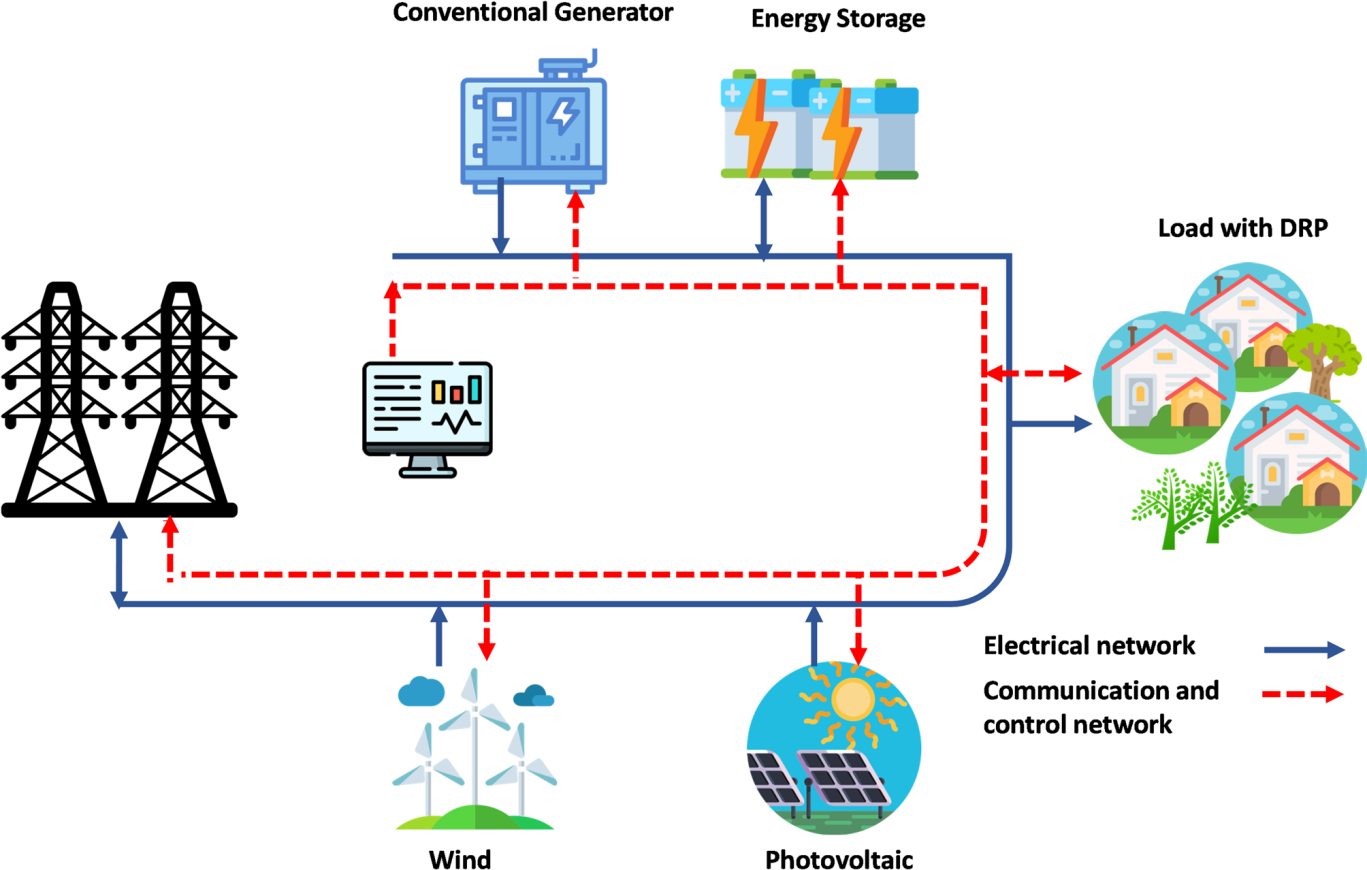
Schematic of a grid-connected MG.

The studied grid-connected MG is shown in Fig. 1, including different types of DERs such as, conventional diesel generators (CDGs), Photovoltaic system, wind turbine system, BESS, and customers with DRP.

### Power transaction model

This paper assumes that the MG and the utility grid (UG) can transact power. If the amount of power transaction with the UG at any given time interval $\:t$ is denoted as $\:{(\mathrm{P}}_{{\mathrm{U}\mathrm{G}}_{\mathrm{t}}}\:\:)$, then the cost of power transaction $\:{\mathrm{C}}_{\mathrm{U}\mathrm{G}}\left({\mathrm{P}}_{{\mathrm{U}\mathrm{G}}_{\mathrm{t}}}\:\right)$ are defined using Locational Marginal Prices (LMP’s) ($\:{{\upgamma\:}}_{\mathrm{t}})$^44^ as:

1 $$\:{C}_{UG}\left({P}_{{UG}_{t}}\:\:\right)=\:{\gamma\:}_{t}\times\:{P}_{{UG}_{t}}\:\:$$

### Generation and load modeling

CDG output power may be adjusted flexibly by the operator. Using the quadratic model, the fuel cost of any CDG$\:\:i$ can be represented as^45^:

2 $$\:{C}_{i}\left({P}_{{i}_{t}}\:\right)={a}_{i}{{p}^{2}}_{{i}_{t}}+{b}_{i}{P}_{{i}_{t}}$$

Where,$\:\:{P}_{{i}_{t}}\:$ is the power generated from the $\:\:{i}^{th}$ generator, $\:{a}_{i}$and $\:{b}_{i}$ represent coefficients of the fuel cost for the $\:{i}^{th}$ conventional generator.

### Demand response model

In the DR formulation, customer participation in the DR program depends on the customer type $\:\theta\:$, which indicates his desire to participate in the DRP. $\:\theta\:\:\in\:\:$[0, 1], 0 for the least willing costumer having a and 1 for the most willing customer. The decision variable $\:{\:\mathrm{P}}_{{\mathrm{c}}_{\mathrm{j},\mathrm{t}}}$ represents the amount of consumption reduction by customer $\:j.$.

Then the customer cost function of customer $\:j$ be represented as quadratic function as^46^:

3 $$\:{\mathrm{C}}_{\mathrm{j}}\left({\uptheta\:},{\mathrm{P}}_{{\mathrm{c}}_{\mathrm{j},\mathrm{t}}}\right)={\mathrm{k}}_{1}{{\mathrm{P}}_{{\mathrm{c}}_{\mathrm{j},\mathrm{t}}}}^{2}+{\mathrm{k}}_{2}{\mathrm{P}}_{{\mathrm{c}}_{\mathrm{j},\mathrm{t}}}(1-{{\uptheta\:}}_{j})$$

Where $\:{\mathrm{k}}_{1}$, and $\:{\mathrm{k}}_{2}$ are cost coefficients.

The customer’s benefit is defined as the difference between the received incentive $\:{\mathrm{y}}_{\mathrm{j},\mathrm{t}}\:$ and the associated cost function can be calculated as follows:

4 $$\:{B}_{1,j}(\theta\:,y,{\mathrm{P}}_{{c}_{j,t}})={y}_{j,t}-({k}_{1}{{\mathrm{P}}_{{c}_{j,t}}}^{2}+{k}_{2}{\mathrm{P}}_{{c}_{j,t}}-{k}_{2}{\mathrm{P}}_{{c}_{j,t}}{{\uptheta\:}}_{j}),\:for\:j\:=\hspace{0.17em}\mathrm{1,2},\dots\:\dots\:J$$

The customers will participate in DRP only in case that their net benefit is non-negative $\:{(\mathrm{B}}_{\mathrm{1,j}}\ge\:0)$.

Then MGO benefit from customer $\:\mathrm{j}$ who participates in DRP is given by:

5 $$\:{\mathrm{B}}_{2,\mathrm{j}}\left({\uptheta\:},{\uplambda\:},{\mathrm{P}}_{{\mathrm{c}}_{\mathrm{j},\mathrm{t}}}\right)={{\uplambda\:}}_{\mathrm{j},\mathrm{t}}{\mathrm{P}}_{{\mathrm{c}}_{\mathrm{j},\mathrm{t}}}-{\mathrm{y}}_{\mathrm{j},\mathrm{t}}$$

Where $\:{{\uplambda\:}}_{\mathrm{j},\mathrm{t}}$ is the cost of power interruption for customer $\:\mathrm{j}$ at time interval $\:\mathrm{t}.$.

Consequently, the overall benefit of MGO is computed based on (5) for the whole interval $\:T$ as:

6 $$\:{\mathrm{B}}_{2}=\sum\:_{\mathrm{j}=1}^{\mathrm{J}}\sum\:_{\mathrm{t}=1}^{\mathrm{T}}{{\uplambda\:}}_{\mathrm{j},\mathrm{t}}{\mathrm{P}}_{{\mathrm{c}}_{\mathrm{j},\mathrm{t}}}-{\mathrm{y}}_{\mathrm{j},\mathrm{t}}\:\:$$

### Storage system modeling

In this paper, the most popular type of storage system in MG, Lithium-ion BESS, is considered.

The BESS unit power at any time interval $\:\mathrm{t}$ is given as^47^:

7 $$\:{P}_{\mathrm{B}\mathrm{E}\mathrm{S}\mathrm{S},\mathrm{t}}={P}_{dis,t}\times\:{b}_{BESS,t}-{P}_{ch,t}\times\:(1-{b}_{BESS,t})\:{b}_{BESS,t}\in\:\left[\mathrm{0,1}\right]$$

Where $\:{b}_{BESS,t}$ is an integer variable used to express the mode of battery (charging/discharging); it has a value of 0 in the charging mode and 1 in the discharging mode; $\:{P}_{ch,t}$ and $\:{P}_{dis,t}$ are the power charged (to the battery) and discharged at any time interval$\:\:t$. Thus, $\:{P}_{\mathrm{B}\mathrm{E}\mathrm{S}\mathrm{S},\mathrm{t}}$ will be positive in discharging and negative at discharging.

Battery State of Charge (SoC) at the end of any interval $\:\mathrm{t}$ ($\:{\mathrm{S}\mathrm{o}\mathrm{C}}_{t+1})\:$can be expressed as^48^:

$$\:\mathrm{C}\mathrm{h}\mathrm{a}\mathrm{r}\mathrm{g}\mathrm{i}\mathrm{n}\mathrm{g}:\:{\mathrm{S}\mathrm{o}\mathrm{C}}_{t+1}=\:{\mathrm{S}\mathrm{o}\mathrm{C}}_{t}+{\eta\:}_{ch}\times\:{P}_{ch,t}\times\:\varDelta\:t\:\:$$

8 $$\:\mathrm{D}\mathrm{i}\mathrm{s}\mathrm{c}\mathrm{h}\mathrm{a}\mathrm{r}\mathrm{g}\mathrm{i}\mathrm{n}\mathrm{g}:\:{\mathrm{S}\mathrm{o}\mathrm{C}}_{t+1}=\:{\mathrm{S}\mathrm{o}\mathrm{C}}_{t}+\frac{{P}_{dis,t}\times\:\varDelta\:t}{{\eta\:}_{dis}}$$

where $\:{\mathrm{S}\mathrm{o}\mathrm{C}}_{t}$ is the battery’s SOC at time $\:t$; $\:{\eta\:}_{ch}$ and $\:{\eta\:}_{dis}$ are the charging and discharging efficiency of BESS.

The cost of utilizing the battery energy storage system is expressed as:

9 $$\:{\mathrm{C}}_{\mathrm{B}\mathrm{E}\mathrm{S}\mathrm{S}}=\:\sum\:_{t=1}^{T}\alpha\:({P}_{dis,t}\times\:{b}_{BESS,t}-{P}_{ch,t}\times\:\left(1-{b}_{BESS,t}\right))\:+{\mathrm{T}\mathrm{C}\mathrm{P}\mathrm{D}}_{\mathrm{B}\mathrm{E}\mathrm{S}\mathrm{S}}\:\:\:{,b}_{BESS,t}\in\:\left[\mathrm{0,1}\right]\:\:\:$$

Where $\:\alpha\:$ is the cost of battery operation in both charge and discharge modes; and $\:{\mathrm{T}\mathrm{C}\mathrm{P}\mathrm{D}}_{\mathrm{B}\mathrm{E}\mathrm{S}\mathrm{S}}\:$is the total cost per day of BESS.

$\:{\mathrm{T}\mathrm{C}\mathrm{P}\mathrm{D}}_{\mathrm{B}\mathrm{E}\mathrm{S}\mathrm{S}}$ is a function of the capital cost and the maintenance cost, and it is given as^48^:

10 $$\:\mathrm{T}\mathrm{C}\mathrm{P}\mathrm{D}=\frac{1}{360}\left[\frac{r(1+r{)}^{LT}}{\left(1+r\right)-1}\times\:\left({C}_{cap}\times\:ES\right)+({C}_{MC}\times\:ES)\right]\:$$

Where $\:r$ is the interest rate (%);$\:\:LT$ is the Battery’s lifetime; $\:{C}_{cap}$, $\:ES$, and $\:{C}_{MC}$ are the capital cost, size, and maintenance cost of BESS, respectively.

## Formulation of the energy management problem

The MG’sconfiguration comprises different types of DER, such as CDG, PV, WT, BESS, and DRP loads. The operating cost is generation, power transactions, and BESS costs. The main objective of the EM is to determine the optimal scheduling for the generation, BESS charging/discharging and load curtailment. The optimal scheduling in the sake of minimizing the operating cost while maximizing the MGO benefit. A multi-objective optimization problem is formulated and solved for this objective using EO. The following section presents a mathematical description of two unique objective functions and their respective constraints.

### Objective function

The formulation of the multi-objective function formulation is as follows:

- The first objective function ($\:{f}_{1}\left(x\right))$ is to minimization of MG’s operating cost. The operating cost can be mathematically expressed as:

11 $$\:{min}{f}_{1}\left(x\right)=min\sum\:_{t=1}^{T}\sum\:_{i=1}^{I}{C}_{i}\left({P}_{{i}_{t}}\:\right)+\sum\:_{t=1}^{T}{C}_{UG}\left({P}_{{UG}_{t}}\:\:\right)\:\:\:+\:\:\:{\mathrm{C}}_{\mathrm{B}\mathrm{E}\mathrm{S}\mathrm{S}}$$

Where $\:I$ is the total number of CDG.

The first term in equation (11) represents the generation cost of CGDs, the second term is the cost of power transaction with the grid, while the third term is the cost of utilizing the BESS in the MG.

b) The second objective function ($\:{f}_{2}\left(x\right)\:)\:$is the maximization of the MGO benefit by incorporating the DRP in the EM for MG. This MGO benefit objective function can be expressed as:

12 $$\:{max}{f}_{2}\left(x\right)=max\sum\:_{j=1}^{J}\sum\:_{t=1}^{T}{\lambda\:}_{j,t}{\mathrm{P}}_{{c}_{j,t}}-{y}_{j,t}\:\:$$

Therefore, the Multi-objective function for MG energy management is represented mathematically using the weighting factors technique as:

13 $$\:{min}{w}_{1}\left[\left.\sum\:_{t=1}^{T}\sum\:_{i=1}^{I}{C}_{i}\left({\mathrm{P}}_{{\mathrm{C}\mathrm{D}\mathrm{G}}_{\mathrm{i},\mathrm{t}}}\:\right)+\sum\:_{t=1}^{T}{C}_{UG}\left({P}_{{UG}_{t}}\:\:\right)\:\:+{\mathrm{C}}_{\mathrm{B}\mathrm{E}\mathrm{S}\mathrm{S}}\:\:\:\:\:\:\:\:\:\:\:\right]\right.+{w}_{2}\:\:\left[\left.\sum\:_{j=1}^{J}\sum\:_{t=1}^{T}{{y}_{j,t}-\lambda\:}_{j,t}{\mathrm{P}}_{{c}_{j,t}}\:\:\right]\right.$$

With the weighting factors Equation should be satisfied as:

14 $$\:{w}_{1}+{w}_{2}=1$$

### Constraints

#### Generation constraints^49^

The power generated and load demand should follow the following equation:

15 $$\:\sum\:_{i=1}^{I}{P}_{{i}_{t}}+{P}_{{UG}_{t}}+{{P}_{w}}_{t}+{{P}_{PV}}_{t}+{P}_{\mathrm{B}\mathrm{E}\mathrm{S}\mathrm{S},\mathrm{t}}={L}_{t}-\sum\:_{j=1}^{J}{\mathrm{P}}_{{c}_{j,t}}.\:\:\:\:\:\:\:\:\:\:\:\:\:\:\:\:\:\:$$

Where $\:{L}_{t}\:$ represents the initial value of load demand at time interval $\:t.$ $\:{{P}_{w}}_{t}$ and $\:{{P}_{PV}}_{t}$ are the power generated from WT and PV respectively.

#### Dispatchable CDGs constraints^49^

The generated power from any CGD $\:i$ should follow the next equations as:

16 $$\:{P}_{{i}_{min}}\le\:{P}_{{i}_{t}}\le\:{P}_{{i}_{max}}$$

17 $$\:{-DR}_{i}\le\:{P}_{{i}_{t+1}}-{P}_{{i}_{t}}\le\:{UR}_{i}$$

Where $\:{P}_{{i}_{min}}$ and $\:{P}_{{i}_{max}}\:$ are the $\:{i}^{th}$CDG minimum and maximum generating limit; $\:{DR}_{i}$ and $\:{UR}_{i}$ are ramp up and down generation rates.

#### Grid power constraint^49^

The power transacted with the UG should be within the maximum permissible limit$\:\:{\mathrm{P}}_{{\mathrm{U}\mathrm{G}}_{\mathrm{m}\mathrm{a}\mathrm{x}}}$as:

18 $$\:{-P}_{{UG}_{max}}\le\:{P}_{{UG}_{t}}\le\:{P}_{{UG}_{max}}$$

#### Demand response program constraints

Constraints for power curtailment of customer $\:\mathrm{j}$ during the study period is:

19 $$\:\sum\:_{t=1}^{T}{\mathrm{P}}_{{c}_{j,t}}\le\:{CM}_{j}$$

Where $\:{\mathrm{C}\mathrm{M}}_{\mathrm{j}}$ is the maximum curtailment ber day for custmer $\:\mathrm{j}$.

The customer will participate in DRP only if his benefit is larger than a; the customer’s benefit is not calculated only as in (4), but it is extended to be for the whole period (24 h); constraint for customer$\:\:j$ benefit is expressed as^49^:

20 $$\:\sum\:_{t=1}^{T}{y}_{j,t}-({k}_{1}{{\mathrm{P}}_{{c}_{j,t}}}^{2}+{k}_{2}{\mathrm{P}}_{{c}_{j,t}}-{k}_{2}{\mathrm{P}}_{{c}_{j,t}}{\theta\:}_{j}\ge\:0$$

Another constraint for DRP is the daily MG budget upper limit ($\:UBL)\:$as^49^:

21 $$\:\sum\:_{t=1}^{T}\sum\:_{j=1}^{J}{y}_{j,t}\le\:UBL$$

#### BESS constraints

The battery SoC limitation is expressed as:

22 $$\:{{\mathrm{S}\mathrm{o}\mathrm{C}}_{min}<\mathrm{S}\mathrm{o}\mathrm{C}}_{t}<{\mathrm{S}\mathrm{o}\mathrm{C}}_{max}$$

Equation (22) determines the minimum $\:{\mathrm{S}\mathrm{o}\mathrm{C}}_{min}$ and maximum $\:{\mathrm{S}\mathrm{o}\mathrm{C}}_{max}\:$power output from BESS. In addition, at any time interval $\:\mathrm{t}$ there are limits for the maximum power charged ($\:{P}_{ch,\mathrm{m}\mathrm{a}\mathrm{x}\_t})$/discharged ($\:{P}_{dis,\mathrm{m}\mathrm{a}\mathrm{x}\_t}$) to/from BESS based on the value of $\:{\mathrm{S}\mathrm{o}\mathrm{C}}.$ These limits are calculated as:

23 $$\:{P}_{ch,\mathrm{m}\mathrm{a}\mathrm{x}\_t}=max\left[\frac{{\mathrm{S}\mathrm{o}\mathrm{C}}_{t+1}-{\mathrm{S}\mathrm{o}\mathrm{C}}_{max}}{\varDelta\:t},{\mathrm{P}}_{min}\right]$$

24 $$\:{P}_{dis,\mathrm{m}\mathrm{a}\mathrm{x}\_t=}min\left[\frac{{\mathrm{S}\mathrm{o}\mathrm{C}}_{t+1}-{\mathrm{S}\mathrm{o}\mathrm{C}}_{max}}{\varDelta\:t},{\mathrm{P}}_{max}\right]$$

where $\:{\mathrm{P}}_{min}$ and $\:{\mathrm{P}}_{max}$ are the installed charging and discharging power.

## Probabilistic operation and uncertainty modeling of MG

This paper considers the uncertainty in RESs generation (PV and wind), energy cost, and load demand. The uncertainties influence MG’s operation and also affect BESS’s sizing process. Therefore, km + 1 PEM with EO optimization technique are used to deal with the uncertainty and solve the sizing problem in the probabilistic framework. Statistical characterization of the random input variable is required for the probabilistic formulation. The output power from WT and PV are random variables because of the random nature of wind speed and solar irradiation. Not only is the RESs generation a random variable, but load demand and energy prices as well^50^.

- Statistical characterization of Solar power

The generated power from PV is expressed as^51^:

25 $$\:{\mathrm{P}}_{\mathrm{P}\mathrm{V}}={\mathrm{P}}_{\mathrm{S}\mathrm{T}\mathrm{C}}\frac{\mathrm{I}}{1000}\left[1+{\upgamma\:}(\mathrm{T}-25)\right]$$

where $\:{\mathrm{P}}_{\mathrm{S}\mathrm{T}\mathrm{C}}$ is the maximum power of PV at slandered condition (irradiation,$\:\mathrm{I}\:$1000 w/m^2^,module temperature $\:\mathrm{T}$= 25 °C); $\:{\upgamma\:}$ is the temperature coefficient of PV module (°C ^− 1^).

In this work, solar irradiance is assumed to follow a normal distribution. Accordingly, the probability density function (PDF) of any random input variable$\:{\mathrm{z}}_{\mathrm{i}}$ is given as:

26 $$\:{\mathrm{f}}_{{\mathrm{z}}_{\mathrm{i}}}\left({\mathrm{z}}_{\mathrm{i}}\right)=\frac{1}{{\upsigma\:}\sqrt{2{\uppi\:}}}.\:{\mathrm{e}}^{-({\mathrm{z}}_{\mathrm{i}}-{\upmu\:}{)}^{2}/2{{\upsigma\:}}^{2}}\:$$

Therefore, the Cumulative Distribution Functions (CDF) is expressed as:

27 $$\:{\mathrm{F}}_{{\mathrm{z}}_{\mathrm{i}}}\left({\mathrm{z}}_{\mathrm{i}}\right)=\frac{1}{2}\left[1+\mathrm{e}\mathrm{r}\mathrm{f}\left(\frac{{\mathrm{z}}_{\mathrm{i}}-{\upmu\:}}{\sqrt{2}{\upsigma\:}}\right)\right]\:\:$$

Then variable $\:{\mathrm{z}}_{\mathrm{i}}$ can be calculated as:

28 $$\:{\mathrm{z}}_{\mathrm{i}}={\upmu\:}+\sqrt{2}{\upsigma\:}.{\mathrm{e}\mathrm{r}\mathrm{f}}^{-1}(2\mathrm{r}-1)\:\:\:$$

With $\:{\upmu\:}$ is variable $\:{\mathrm{z}}_{\mathrm{i}}$ mean value, the error function $\:\left(\mathrm{e}\mathrm{r}\mathrm{f}\right)$ and its inverse $\:\left({\mathrm{e}\mathrm{r}\mathrm{f}}^{-1}\right)$ can be expressed as:

29 $$\:\mathrm{e}\mathrm{r}\mathrm{f}\left({\mathrm{z}}_{\mathrm{i}}\right)=\frac{2}{\sqrt{{\uppi\:}}}{\int\:}_{0}^{\mathrm{z}}{\mathrm{e}}^{{-\mathrm{t}}^{2}}\mathrm{d}\mathrm{t}\:\:$$

30 $$\:{\mathrm{erf}}^{-1}\left({\mathrm{z}}_{\mathrm{i}}\right)=1-\mathrm{e}\mathrm{r}\mathrm{f}\left(\mathrm{z}\right)$$

- Statistical characterization of Wind power

The generated power from WT can be expressed as:

31 $$\:{\mathrm{P}}_{\mathrm{w}}=\left\{\begin{array}{c}0\:\:\:\:\:\:\:\:\:\:\:\\\:\frac{{\mathrm{v}}^{2}-{\mathrm{v}}_{\mathrm{c}\mathrm{i}}^{2}}{{\mathrm{v}}_{\mathrm{n}\mathrm{o}\mathrm{m}}^{2}-{\mathrm{v}}_{\mathrm{c}\mathrm{i}}^{2}}.\\\:{\mathrm{P}}_{\mathrm{n}\mathrm{o}\mathrm{m}}\end{array}\right.{\mathrm{P}}_{\mathrm{n}\mathrm{o}\mathrm{m}}\:\:\:\:\:\:\:\:\:\genfrac{}{}{0pt}{}{\begin{array}{c}v\le\:{\mathrm{v}}_{\mathrm{c}\mathrm{i}}\:and\:\:v\ge\:{\mathrm{v}}_{\mathrm{c}\mathrm{o}}\\\:\:\end{array}}{\begin{array}{c}{\mathrm{v}}_{\mathrm{c}\mathrm{i}}\le\:v\le\:{\mathrm{v}}_{\mathrm{n}\mathrm{o}\mathrm{m}}\:\:\\\:\:\\\:{\mathrm{v}}_{\mathrm{n}\mathrm{o}\mathrm{m}}<v\le\:{\mathrm{v}}_{\mathrm{c}\mathrm{o}}\end{array}}$$

where,$\:\:{\mathrm{v}}_{\mathrm{c}\mathrm{i}}$ and $\:{\mathrm{v}}_{\mathrm{c}\mathrm{o}}$ are the cut-in and cut-out wind speed, the rated power of WT, $\:{\mathrm{v}}_{\mathrm{n}\mathrm{o}\mathrm{m}}\:$and $\:{\mathrm{P}}_{\mathrm{n}\mathrm{o}\mathrm{m}}$ are the rated wind speed and power, and $\:\mathrm{v}\:$ is wind speed.

To express the PDF of the WT speed, a Weibull distribution is used as^52^:

32 $$\:\:\:\:\:\:\:\:{\mathrm{f}}_{\mathrm{v}}\left(\mathrm{v}\right)=\left\{\begin{array}{c}0,\:\:\:\:\:\:\:\:\:\:\:\:\:\:\:\:\:\:\:\:\:\:\:\:\:\:\:\:\:\:\:\:\:\:\:\:\:\:\:\:v<0\\\:\frac{\mathrm{k}}{\mathrm{C}}.{\left(\frac{\mathrm{v}}{\mathrm{C}}\right)}^{\mathrm{k}-1}\:.{\mathrm{e}}^{{\left(\frac{\mathrm{v}}{\mathrm{C}}\:\right)}^{\mathrm{k}}},\:\:v\ge\:0\end{array}\right.$$

The CDF can be obtained as:

33 $$\:\:\:\:\:\:\:\:\:\:\:\:\:\:\:{\mathrm{F}}_{\mathrm{v}}\left(\mathrm{v}\right)=\left\{\begin{array}{c}0,\:\:\:\:\:\:\:\:\:\:\:\:\:\:\:\:\:\:\:\:\:\:\:\:\:\:\:\:\:v<0\\\:1-{\mathrm{e}}^{{-\left(\frac{\mathrm{v}}{\mathrm{C}}\:\right)}^{\mathrm{k}}},\:\:v\ge\:0\end{array}\right.\:\:\:\:\:\:\:\:$$

Then wind speed is computed as following:

34 $$\:\:\:\:\:\:\:\:\:\:\:\:\:\:\:\:\:\:\:\mathrm{v}=\mathrm{C}.{\left(-\mathrm{ln}\left(\mathrm{r}\right)\right)}^{\raisebox{1ex}{$1$}\!\left/\:\!\raisebox{-1ex}{$\mathrm{k}$}\right.}\:\:\:\:\:\:\:\:\:\:\:$$

The scale (C) and the shape (K) parameters Weibull distribution can be calculated using different methods^53, 54^. Using Standard Deviation (STD) $\:{\upsigma\:}$ and mean wind speed, the parameters are approximately calculated as^53^:

35 $$\:\:\:\:\:\:\:\:\:\:\:\:\:\:\:\:\:\:\:\:\:\:\:\:\:\mathrm{k}={\left(\frac{{\upsigma\:}}{{\mathrm{v}}_{\mathrm{m}}}\right)}^{-1.086}\:\:\:\:\:\:\:\:\:\:\:\:\:$$

36 $$\:\:\:\:\:\:\:\:\mathrm{C}=\frac{{\mathrm{v}}_{\mathrm{m}}}{{\Gamma\:}(1+\raisebox{1ex}{$1$}\!\left/\:\!\raisebox{-1ex}{$\mathrm{k}$}\right.)}\:\:\:\:\:\:\:\:$$

The gamma function ($\:{\Gamma\:}\:\left(\mathrm{x}\right)$) is calculated as:

37 $$\:\:\:\:\:\:\:\:\:\:\:\:\:\:\:\:\:\:\:{\Gamma\:}\:\left(\mathrm{x}\right)={\int\:}_{0}^{{\infty\:}}{\mathrm{t}}^{\mathrm{x}-1}.{\mathrm{e}}^{-\mathrm{t}}\mathrm{d}\mathrm{t}\:\:\mathrm{f}\mathrm{o}\mathrm{r}\:\:\:\:\mathrm{x}>0\:\:\:\:\:\:\:\:\:\:$$

- Statistical characterization of Load and energy prices

The uncertainties in load and energy prices are modeled using a normal probability density function (PDF)^55^. Accordingly, for any random variable $\:{}_{}{\mathrm{L}}_{\mathrm{i}}$ the PDF can be expressed as:

38 $$\:{f}_{{L}_{i}}\left({L}_{i}\right)=\frac{1}{\sigma\:\sqrt{2\pi\:}}.\:{e}^{-({L}_{i}-\mu\:{)}^{2}/2{\sigma\:}^{2}}\:$$

where $\:{\upmu\:}$ and $\:{\upsigma\:}$ are the mean and standard division (SD) values respectively for variable $\:{\mathrm{L}}_{\mathrm{i}}$.

### Point estimation method

PEM, one of the approximations methods, employs a deterministic technique for solving probabilistic problems. PEM offers a great reduction in computational efforts in comparison to MCS. The idea of PEM was first proposed in 1975 by Rosenblueth^56^. Then Hong, in 1998, proposed a technique where the simulation number is linearly proportional to the number of random variables^57^.

#### Point estimation method (PEM)

PEMs are employed to linearize the output variables concerning the input random variable (IRV). In PEM, using the $\:\mathrm{m}$ (number of IRV), the moments of output variable $\:\mathrm{y}$ are computed i.e. $\:\:\:\mathrm{y}=\mathrm{F}({\mathrm{p}}_{1},{\mathrm{p}}_{2},\dots\:\dots\:,{\mathrm{p}}_{\mathrm{m}})$. For each IRV, the forecasted data of $\:\mathrm{m}$ input random variable are used to concentrate the first few moments on $\:\:\mathrm{k}=\mathrm{2,3},5,\dots\:.$ Points (central moment’s points). Then the statistical moments of $\:\mathrm{y}$ are evaluated a number of times based on the PEM’s scheme $\:2\mathrm{m}$, $\:2\mathrm{m}+1$, or $\:4\mathrm{m}+1$. $\:2\mathrm{m}+1$ method is more accurate than $\:2\mathrm{m}$, and its performance is nearly the same as $\:4\mathrm{m}+1$^58, 59^. So in this paper, $\:2\mathrm{m}+1$ scheme is adopted.

For each variable of IRV, $\:2\mathrm{m}+1\:$ scheme use three points or standard locations, one of which is the mean value. An expression for the standard locations using the skewness $\:{{\uplambda\:}}_{\mathrm{t},3}$ (third moment), and kurtosis $\:{{\uplambda\:}}_{\mathrm{t},4}$ (fourth standard moment) of $\:{\mathrm{p}}_{\mathrm{t}}\:$ IRV as:

39 $$\:{{\upxi\:}}_{\mathrm{t},\mathrm{k}}=\frac{{{\uplambda\:}}_{\mathrm{t},3}}{2}+(-1{)}^{3-\mathrm{k}}.\sqrt{{{\uplambda\:}}_{\mathrm{t},4}-\frac{3}{4}}{{{\uplambda\:}}^{2}}_{\mathrm{t},3}\:\:\:\:\:\mathrm{k}=\mathrm{1,2}\:\mathrm{a}\mathrm{n}\mathrm{d}\:{{\upxi\:}}_{\mathrm{t},3}=0$$

Then variable $\:{\mathrm{p}}_{\mathrm{t}}$ locations are calculated as:

40 $$\:\:\:\:\:\:\:\:\:\:\:\:\:\:{\:\mathrm{p}}_{\mathrm{t},\mathrm{k}}\:={{\upmu\:}}_{{\mathrm{p}}_{\mathrm{t}}}+{{\upxi\:}}_{\mathrm{t},\mathrm{k}}.\:{{\upsigma\:}}_{{\mathrm{p}}_{\mathrm{t}}}\:\:\:\:\:\:\mathrm{k}=\mathrm{1,2},3\:\:\:\:$$

Then the weight is calculated for each location as follows:

41 $$\:{\mathrm{w}}_{\mathrm{t},\mathrm{k}}\:=\left\{\begin{array}{c}\frac{{(-1)}^{3-\mathrm{k}}}{{{\upxi\:}}_{\mathrm{t},\mathrm{k}}({{\upxi\:}}_{\mathrm{t},1}-{{\upxi\:}}_{\mathrm{t},2})}\:\:\:\:\:\:\:if\:k=\mathrm{1,2}\\\:\frac{1}{\mathrm{m}}-\frac{1}{{{\uplambda\:}}_{\mathrm{t},4}-{{{\uplambda\:}}^{2}}_{\mathrm{t},3}}\:\:\:\:\:\:\:\:\:\:\:\:\:\:k=3\end{array}\right.$$

For all IRV, setting $\:{{\upxi\:}}_{\mathrm{t},\mathrm{k}}$ in (40) leads to $\:{\:\mathrm{p}}_{\mathrm{t},\mathrm{k}}={{\upmu\:}}_{{\mathrm{p}}_{\mathrm{t}}}$, which is the mean value of IRVs. So the output $\:\mathrm{y}\:$will be evaluated once for all IRVs at $\:{({\upmu\:}}_{{\mathrm{p}}_{1}},{{\upmu\:}}_{{\mathrm{p}}_{2}},\dots\:,{{\upmu\:}}_{{\mathrm{p}}_{\mathrm{t}}},...,{{\upmu\:}}_{{\mathrm{p}}_{\mathrm{m}}})$and the weight is calculated as:

42 $$\:\:\:\:\:\:\:\:\:\:\:\:\:\:{\mathrm{w}}_{0}\:=1-\sum\:_{\mathrm{t}=1}^{\mathrm{m}}\frac{1}{{{\uplambda\:}}_{\mathrm{t},4}-{{{\uplambda\:}}^{2}}_{\mathrm{t},3}}\:\:\:\:\:\:\:\:\:\:\:\:$$

For each IRV the moments’ vector of $\:\mathrm{y}$ are evaluated as:

43 $$\:\:\:\:\:\:\:\:\:\:\:\mathrm{E}\left({\mathrm{Y}}^{\mathrm{j}}\right)=\mathrm{E}\left({\mathrm{Y}}^{\mathrm{j}}\right)+\sum\:_{\mathrm{i}=1}^{2}{\mathrm{w}}_{\mathrm{i},\mathrm{t}}.{\left[\mathrm{F}\left({\mathrm{P}}_{\mathrm{i}}\right)\right]}^{\mathrm{j}}\:\:\:\:\:\:\:\:\:\:\:\:\:$$

And after evaluation $\:\mathrm{y}$ using the mean values, the output moment vector is updated as:

44 $$\:\:\:\:\:\:\:\:\:\:\:\mathrm{E}\left({\mathrm{Y}}^{\mathrm{j}}\right)=\mathrm{E}\left({\mathrm{Y}}^{\mathrm{j}}\right)+{\mathrm{w}}_{0}{\left[\mathrm{F}({{\upmu\:}}_{\mathrm{p})}\right]}^{\mathrm{j}}\:\:\:\:\:\:\:\:\:\:\:$$

Then the mean $\:\left({{\upmu\:}}_{\mathrm{Y}}\right)\:$and STD ($\:{{\upsigma\:}}_{\mathrm{Y}})\:$of $\:\mathrm{y}$ can be evaluated as:

45 $$\:\:\:\:\:\:\:\:\:\:\:{{\upsigma\:}}_{\mathrm{Y}}=\sqrt{\mathrm{E}\left({\mathrm{Y}}^{2}\right)-{{{\upmu\:}}^{2}}_{\mathrm{Y}}\:}\:\:\:\:\:\:\:\:\:\:$$

46 $$\:{{\upmu\:}}_{\mathrm{Y}}=\mathrm{E}\left(\mathrm{Y}\right)\:\:\:\:\:$$

After that PDF of the output function can be estimated using Gram–Charlier series^59^.

## The probabilistic battery energy storage sizing approach

The multi-objective optimal BESS sizing to consider the input variable’s uncertainty is a complex optimization problem. Solving this multi-objective optimization problem imposes different system constraints and BESS limitations. Generally, the optimal size for BESS is chosen after evaluating the total cost for finite numbers of sizes. The size that yields the lowest cost is then selected as the optimal size. This is valid in case of the input variables have a deterministic characteristic. Some of the input variables are characterized by significant levels of uncertainty. This implies treating such variables as random input variables, necessitating a probabilistic method for estimating the size of the BESS. The probabilistic BESS (P-BESS) sizing is solved using a hybrid 2m + 1 PEM with a metaheuristic optimization algorithm named Equilibrium Optimize (EO-PEM). Each IRV is treated with its corresponding PDF. The problem is solved for evaluating the output random variable.

**Fig. 2:**
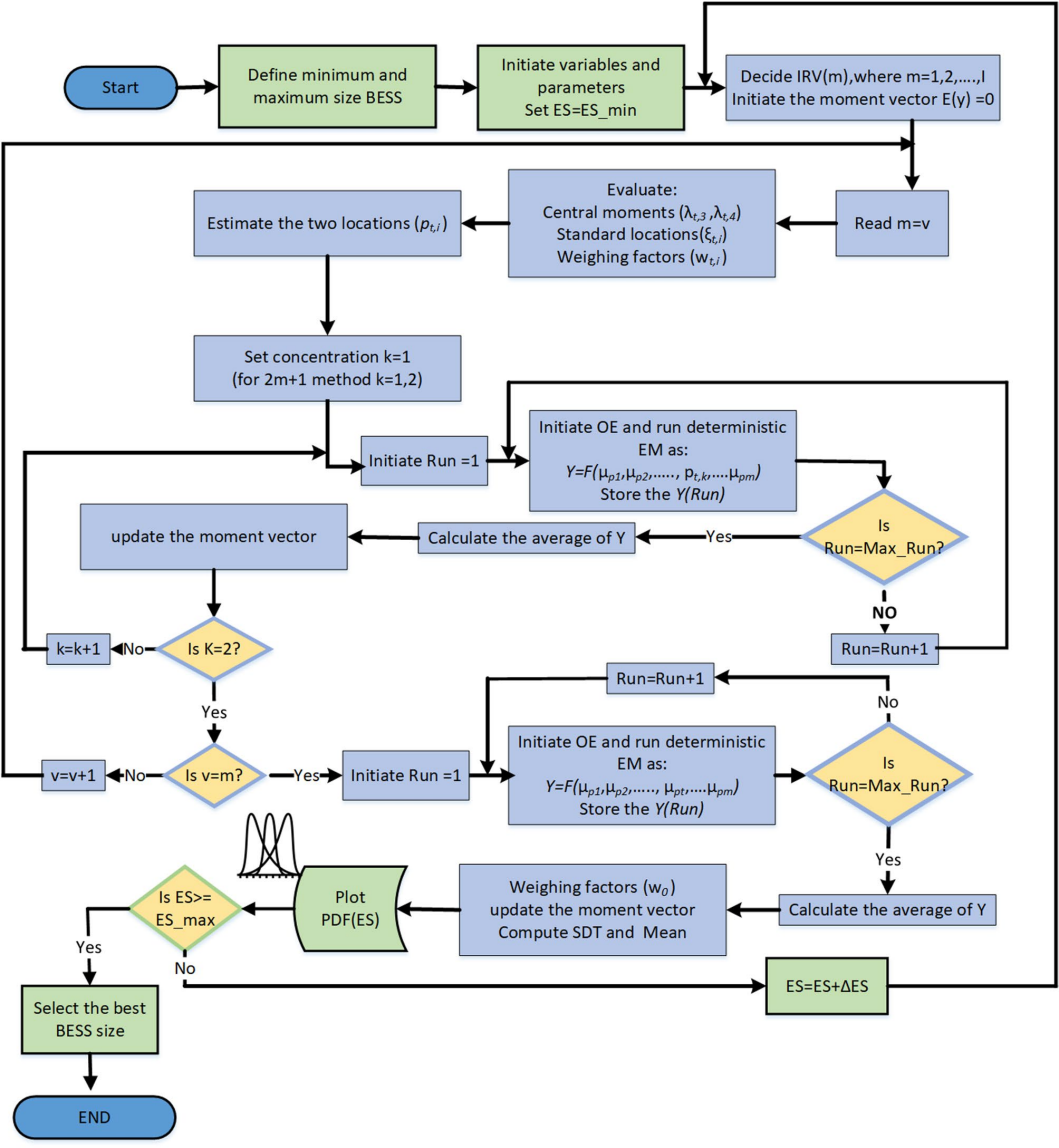
Flowchart of the EO-PEM for P-BESS sizing.

In this paper, the IRVs are the solar PV generation, WT generation, load demand, and energy prices (the equivalent here is the cost of interruption). The flowchart of the EO-PEM for P-BESS is shown in Figure 2. Thepresented sizing technique can be divided into four main stages as follows:

- Stage 1: Start by setting the limit of minimum (ES_min) and maximum (ES_max) size of BESS.
- Stage 2: EO-PEM is used to solve the problem of EM to get the statistical expression of the operating cost function. The EO-PEM stage can be summarized as the following steps.
  - Step 1: the input random variables are defined (v = 1,2,,….,m).
  - Step 2: initiate the moments’ vector of the output variable $\:\mathrm{E}\left({\mathrm{y}}^{\mathrm{j}}\right)=0$. And initiate v = 1 for the first IRV.
  - Step 3: read the v^th^ IRV.
  - Step 4: get the central moments, evaluate the standard location $\:{({\upxi\:}}_{\mathrm{t},\mathrm{k}})$, and weighting factors $\:{(\mathrm{w}}_{\mathrm{t},\mathrm{k}})$.
  - Step 5: estimate variable v locations ($\:{\:\mathrm{p}}_{\mathrm{t},\mathrm{k}}$).
  - Step 6: using EO the problem is solved deterministically for the two positions; also, to attenuate the probabilistic nature of optimization algorithms, the problem is solved for different numbers of runs, and the average value is calculated.
  - Step 7: update the moments’ vector $\:E\left({Y}^{j}\right)$.
  - Step 8: increment v = v + 1 and repeat Steps 3–7 until all IRV are taken.
  - Step 9: solving the deterministic EM problem based on the mean values of the input variable $\:{({\upmu\:}}_{{\mathrm{p}}_{1}},{{\upmu\:}}_{{\mathrm{p}}_{2}},\dots\:,{{\upmu\:}}_{{\mathrm{p}}_{\mathrm{t}}},\dots\:,{{\upmu\:}}_{{\mathrm{p}}_{\mathrm{m}}})$, and calculate the corresponding weight factor $\:{\mathrm{w}}_{0}.$.
  - Step 10: Update the final moments’ vector $\:E\left({Y}^{j}\right)$.
  - Step 11: evaluate mean $\:\left({{\upmu\:}}_{\mathrm{Y}}\right)\:$and STD ($\:{{\upsigma\:}}_{\mathrm{Y}})$.
- Stage 3: After the EO-PEM stage is finished, the PDF of the BESS size is plotted and stored. Then the $\:ES$ is incremented by $\:\varDelta\:ES$.
  The previous stages, 1–3, will be repeated until the maximum size of ES is reached.
- Stage 4: all PDFs are compared, and the optimal size of BESS is selected.

## Solution method

The Equilibrium optimizer is a metaheuristic optimization algorithm developed by Faramarzi, Afshin., et al. in^43^. This algorithm is inspired by the equilibrium and dynamic states in controlling the mass balance. The concentration of non-reactive constituents for the control volume is described by the mass equation. The mass balance equation provides the physical fundamental for the conservation of mass entering, leaving, and being generated in a control volume.

As with most metaheuristic optimization, the optimization process starts with random initialization for the population. Where particles and concentrations are the solutions and positions.

The populations ($\:N$) are initialized based on the lower limit $\:{(C}_{min}$) and upper limits ($\:{C}_{max}$) as:

47 $$\:{C}_{i}^{initial}={C}_{min}+rand*\left({C}_{max}-{C}_{min}\right)\:\:\:i=\mathrm{1,2},\dots\:\dots\:,N$$

This algorithm aims to find the equilibrium point (global optima point). At the beginning of the search process, the equilibrium point is unknown; only equilibrium candidates are used to determine the search pattern. These equilibrium candidates are the best four particles identified through the whole optimization process; the fifth particle, whose concentration is the arithmetic mean of the best four particles, is also used. These entire five particles are used to construct which called the equilibrium pool as follows:

48 $$\:{C}_{eq}^{pool}={{\{C}_{eq}}^{\left(1\right)},{{C}_{eq}}^{\left(2\right)},{{C}_{eq}}^{\left(3\right)},{{C}_{eq}}^{\left(4\right)},{{C}_{eq}}^{\left(avg\right)}\}$$

In each iteration, the concentration for each particle is updated based on random selection from the equilibrium candidates with equal probability. The pool concentration particles are used to update the concentrations for all particles using random candidates at each iteration. For instance the first iteration, all particles concentrations may be updated based on the first equilibrium candidate ($\:{{C}_{eq}}^{\left(1\right)}$), in the next iteration $\:{{C}_{eq}}^{\left(avg\right)}$ may be used to update the concentration; this will continue until the end of the optimization process.

Another exponential term is used in the updating process, which gives EO the balance between exploration and exploitation. This term is expressed as:

49 $$\:Z={e\:}^{\lambda\:(t-{t}_{0})}\:\:$$

Where $\:t$ and $\:{t}_{0}$ are given as:

50 $$\:t=(1-\frac{iter}{\mathrm{m}\mathrm{a}\mathrm{x}\_iter}{)}^{{d}_{2}\left(\raisebox{1ex}{$iter$}\!\left/\:\!\raisebox{-1ex}{$\mathrm{m}\mathrm{a}\mathrm{x}\_iter$}\right.\right)}$$

51 $$\:{t}_{0}=\frac{1}{\lambda\:}In\left(-{d}_{1}sign\left(r-0.5\right)\left[1-{e}^{-\lambda\:t}\right]\right)+t$$

Where $\:iter$ and $\:\mathrm{m}\mathrm{a}\mathrm{x}\_iter$ are the current and maximum iteration; $\:{d}_{1}$ and $\:{d}_{2}$ are two constants to control the exploration and the exportation, respectively; $\:sign\left(r-0.5\right)$ are control the direction of exploration and exploitation; $\:\lambda\:$ and $\:r$ are random vectors in the interval of [0, 1].

The generation rate is another important term that helps in improving the exploitation of the studied algorithm; this term can be expressed as:

52 $$\:GR={GR}_{0}{e\:}^{\lambda\:(t-{t}_{0})}\:\:$$

Where:

53 $$\:{GR}_{0}=GCP({{{C}_{eq}}^{\:}-\lambda\:C)\:}^{\:}\:\:$$

54 $$\:GCP=\left\{\begin{array}{c}0.5{r}_{1}\:\:\:\:\:\:\:\:\:\:{r}_{2}\ge\:GP\\\:0\:\:\:\:\:\:\:\:\:\:\:\:\:\:\:\:{r}_{2}<GP\end{array}\right.$$

Where $\:{r}_{1}$ and $\:{r}_{2}$ are two randomly generated variables, and $\:GP$ is the generation probability.

Then the updating role can be expressed as:

55 $$\:C={C}_{eq}+\left(C-{C}_{eq}\right).Z+\:\frac{GR}{\lambda\:V}(1-Z)$$

The first term in Eq. (55) is the equilibrium concentration, the second for the global searching or to enhance the exploration of the technique, while the third is to enhance the accuracy of the solution.

## Simulation results

To demonstrate the effectiveness and feasibility of the EO-PEM for P-BESS, the proposed sizing technique is employed to solve the energy management problem in MG, a grid-connected MG test system is simulated on i7- 2.9-GHz with RAM of 8-GB using MATLAB 2021b software. In this paper, the MG comprises renewable DER such as PV and wind; dispatchable CGDs; BESS; and customers participating in the DRP. A typical illustration for the studied MG configuration is shown in Fig. 1. At first, the MG is assumed not to have the BESS. MG consists of a PV unit, a WT unit, three CDG units, and three different customers participating in demand response program. Hourly load demand, generated power from PV and WT units, customers power inturabtibilty value are shown in Table 2. Solar radiation data for a site in Harare, Zimbabwe, is calculated stochastically^50^. The wind speed data used in this study are obtained at an altitude of 1480 m above sea level, with measurements taken at an anemometer height of 10 m^50^. The CDG data and customers’ data are listed in Table 3. The daily budget (UBL) of MG is assumed to be a $ 500^49^.

**Table 2:** Power interruptibility λ_j, t_ data for case study I.

Time (h)	Total demand(KW)	Solar (KW)	Wind (KW)	$$\:{\lambda\:}_{1,t}$$ ($)	$$\:{\lambda\:}_{2,t}$$_t_($)	$$\:{\lambda\:}_{3,t}$$ ($)
t = 1	31.83	0	7.56	1.57	3.70	2.70
t = 2	31.4	0	7.5	1.4	2.70	1.90
t = 3	31.17	0	8.25	2.2	3.20	1.80
t = 4	31	0	8.48	3.76	2.60	1.90
t = 5	31.17	0	8.48	4.5	3.80	2.30
t = 6	32.1	0	9.42	4.7	1.70	0.70
t = 7	32.97	0	9.82	5.04	2.30	1.40
t = 8	34.1	7.99	10.35	5.35	1.50	0.50
t = 9	37.53	10.56	10.88	6.7	4.30	2.90
t = 10	38.33	13.61	11.01	6.16	4.60	1.60
t = 11	40.03	14.97	10.94	6.38	3.50	4.30
t = 12	41.17	15	10.68	6.82	4.20	4.80
t = 13	39.67	14.78	10.42	7.3	4.30	5.10
t = 14	41.7	14.59	10.15	7.8	6.30	5.40
t = 15	42.1	13.56	9.67	8.5	3.50	5.50
t = 16	41.67	11.83	8.98	7.1	5.30	6.10
t = 17	40.7	10.17	8.37	6.8	5.30	5.60
t = 18	40.07	7.66	7.61	6.3	6.10	6.30
t = 19	38.63	0	6.7	5.8	2.60	4.50
t = 20	36.4	0	5.72	4.2	3.60	4.20
t = 21	34.1	0	7.21	3.8	4.20	3.90
t = 22	32.8	0	7.75	3.01	3.80	3.20
t = 23	32.5	0	7.88	2.53	2.30	2.80
t = 24	32	0	7.69	1.42	3.80	4.20

**Table 3:** Conventional generators and customers data.

i, j	Conventional generators							Customer		
	a_i_($/MW^2^h)	b_i_($/KW h)	*P*_i, min_ (KW)	*P*_i, max_(KW)	DR_i_ (KW/h)	UR_i_ (KW/h)	$$\:\theta\:$$	K_1,j_	K_2,j_	*CM*_*j*_(KW)
1	0.06	0.5	0	4	3	3	0	1.079	1.32	30
2	0.03	0.25	0	6	5	5	0.45	1.378	1.36	35
3	0.04	0.3	0	9	8	8	0.9	1.847	1.64	40

### Case I: EM problem without BESS

**Fig. 3:**
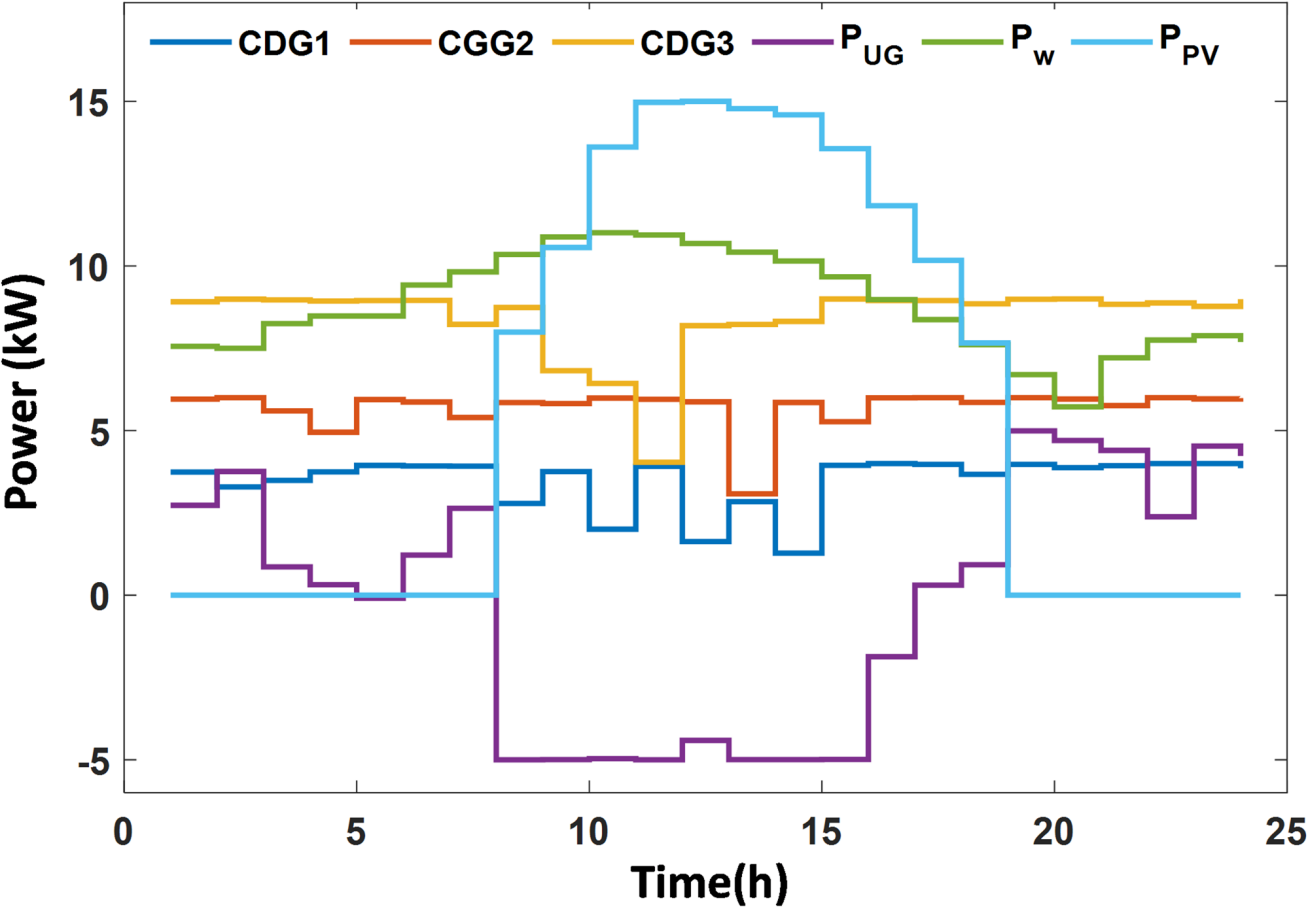
MG generation resources for case I.

**Fig. 4:**
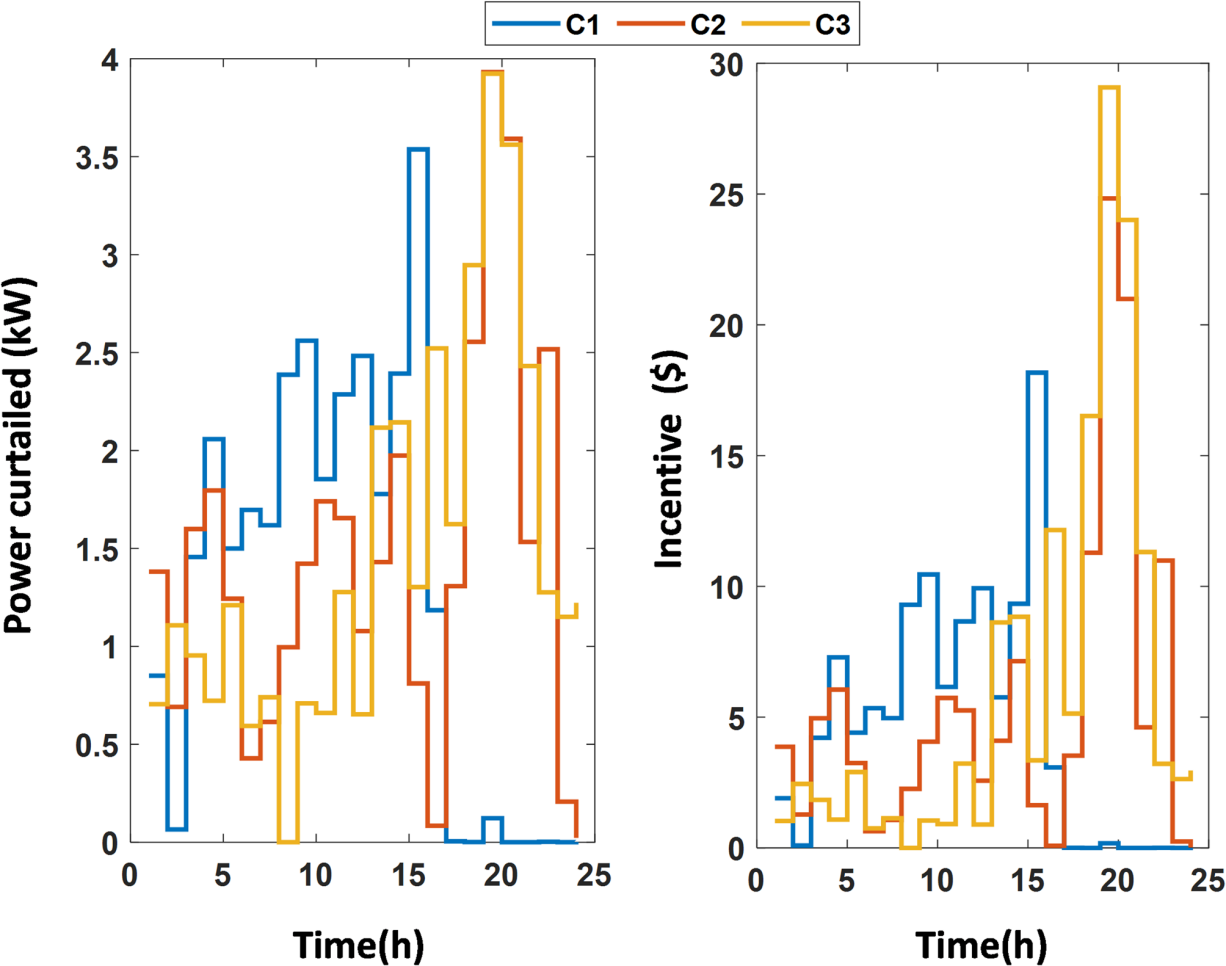
Customers’ curtailment and saving.

In this case study, the effectiveness of the Equilibrium Optimizer (EO) for solving the energy management (EM) problem with the incorporation of demand response programs (DRP) is demonstrated. The microgrid (MG) model is simulated, and the EM problem is solved using various optimization algorithms, including PSO^60^, JAYA^61^, AVOA^62^, INFO^63^, and BWO^64^. Thirty independent runs were performed for each algorithm, and the results are compared in Table 4. Analysis of these results indicates that the EO algorithm outperforms the other methods in solving the deterministic EM problem. Using EO, the generated power from conventional diesel generators (CDGs), photovoltaic (PV) systems, wind turbines (WT), and power transactions with the main grid are shown in Figure 3. The customers’ usage reductions and the corresponding incentive payments are presented in Figure 4.

**Table 4:** Comparison of the EM problem for case I.

	Total operating cost ($)		
Technique	Worst	Best	Mean
PSO^60^	511.04	300.88	395.8729
JAYA^61^	494.20	355.18	431.3128
AVOA^62^	754.58	220.29	301.1224
INFO^63^	737.92	402.69	511.47
BWO^64^	612.32	331.87	443.366
EO	371.93	229.22	296.7653

### Case II: EM problem with BESS sizing

**Fig. 5:**
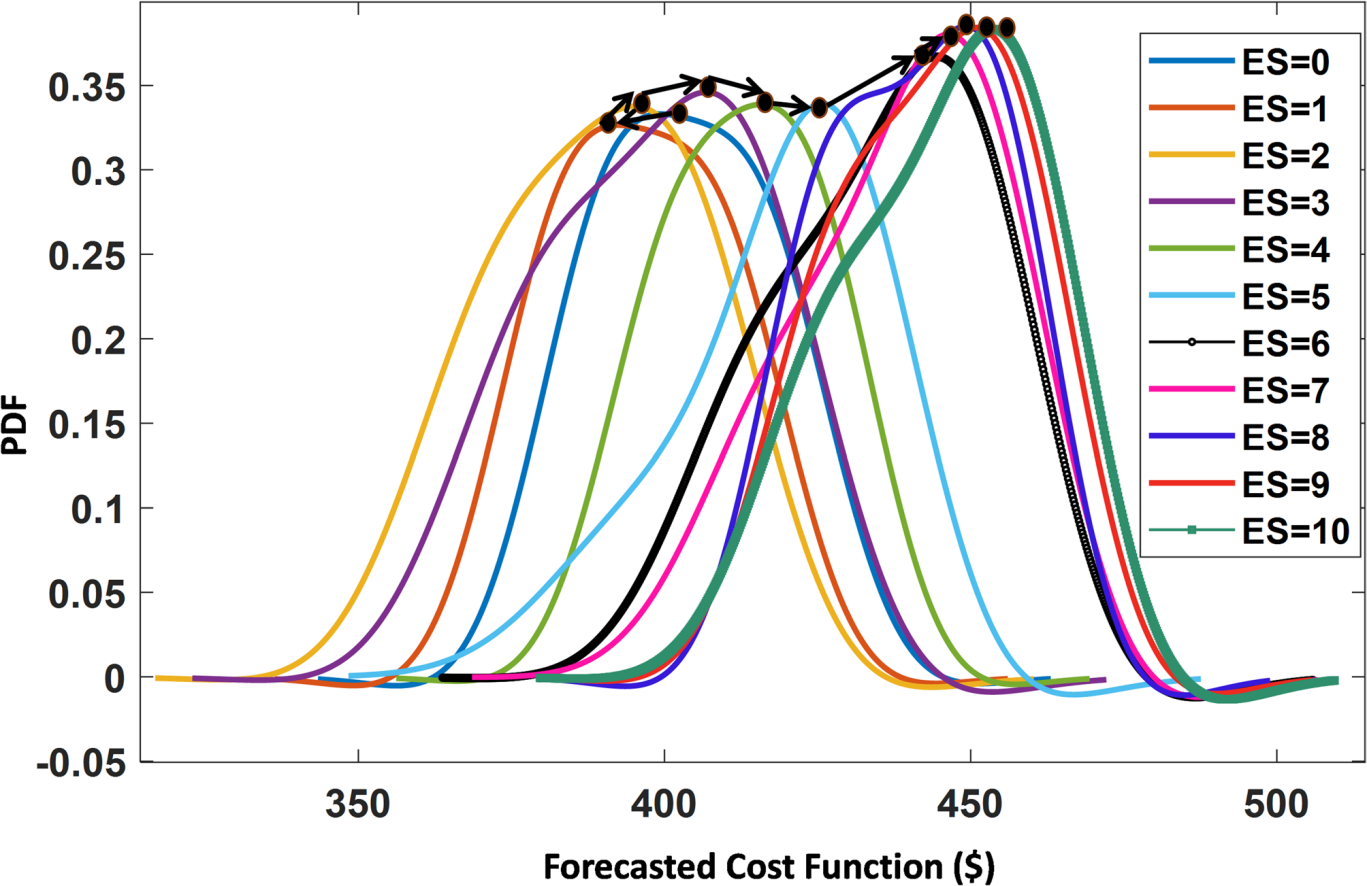
The PDF for the forecasted operating cost for different BESS sizes.

In this case study, a probabilistic sizing for BESS is performed. The presented P-BESS with EO-PEM is employed to get the optimal sizing. The BESS data used in this paper are presented in Table 5. The uncertainty in the generated power from WT and PV, load demand, and Energy prices are considered as the IRVs. With reference for the proper PDFs for each random variable, the probabilistic problem is solved. It is assumed that the WT generation has a Weibull distribution with an STD of 5%; PV, energy prices, and load demand are assumed to have a normal distribution with a STD of 5% for PV and energy prices and 3% for load^25^. In this paper, the size of BESS is considered to start from 0 (No BESS) to 10 kWh,as the studied microgrid is a small-scale system with low peak demand. Therefore, BESS capacities exceeding 10 kWh would be economically unjustifiable and inconsistent with the system’s rated capacity. Using the EO-PEM (2m + 1), the statistical moments are calculated for each size of BESS (as described in section 5). The PDFs of the output variable whiche is the operating cost are shown in Fig. 5.

**Table 5:** BESS parameters.

Parameter	Value
$$\:{\eta\:}_{ch}$$,$$\:\:{\eta\:}_{dis}$$	95%
$$\:{\mathrm{S}\mathrm{O}\mathrm{C}}_{min}$$	10%
$$\:{\mathrm{S}\mathrm{O}\mathrm{C}}_{max}$$	90%
$$\:{\mathrm{S}\mathrm{O}\mathrm{C}}_{0}$$	30%
$$\:r$$	6%
$$\:LT$$	3
$$\:\alpha\:$$	4 ($/MWh)
$$\:{C}_{cap}$$	400 ($/kwh)
$$\:{C}_{MC}$$	30 ($/kwh)

Figure 5 shows that the mean value of the operating cost starts decreasing from no-BESS until the minimum value (ES = 1 kWh) is reached. After this point, the mean value of the operating cost starts to increase again with increasing the BESS size. The low value of the coefficient of the variant and the great resemblance between the coefficients of the variant for the PDF of the operating cost for the different BESS sizes validate the use of mean value as an appropriate variable for selecting BESS size. So by investigating the PDF in Fig. 5, the optimal size of BESS for this case study is selected to be ES = 1 kWh.

## Conclusions

This paper has presented the application of the Equilibrium Optimizer (EO) algorithm to address the energy management (EM) problem in a microgrid (MG), considering uncertainties in MG resources and the demand response (DR) program. The objective of optimal MG operation is to minimize operational costs, including generation costs, power transactions, and the daily total cost of the BESS, while maximizing the Microgrid Operator’s (MGO) benefit when DR is integrated into the EM problem. For the deterministic EM problem, various optimization techniques are employed to solve the day-ahead EM without considering BESS integration. Simulation results indicate that the EO algorithm outperforms other techniques.

The main contribution of this study is the development of an effective probabilistic technique for sizing battery energy storage, which accounts for uncertainties in PV and wind turbine (WT) generation, load demand, and energy prices. The optimal BESS capacity is determined using the 2m + 1 Point Estimation Method (PEM). Uncertainties in the MG are treated as input random variables described by their probability density functions (PDFs): normal distributions for PV generation, load demand, and energy prices, and a Weibull distribution for WT generation. The presented sizing technique is validated through simulation of a MG test system to determine the optimal BESS size. Future work will explore incorporating additional sources of uncertainty, such as electric vehicle charging, market price fluctuations, and equipment failures, to further enhance the robustness of the EO–PEM BESS sizing framework.

## Data Availability

All data generated or analyzed during this study are included in this published article.

## List of symbols

BEES: Battery energy storage system
MG: Microgrid
PEM: Point Estimation Method
EO: Equilibrium optimizer
IDR: Incentive-based Demand Response
EM: Energy management
DER: Distributed energy resources
RES: Renewable energy resources
PV: Photovoltaic
WT: Wind turbine
PSO: Particle swarm optimization
ADMM: Alternating Direction Method of Multipliers
MMG: Multi-Microgrid
MGO: Microgrid operator
MILP: Mixed integer linear programing
SoC: State of Charge
DR: Demand response
MCS: Monte Carlo Simulation
CDG: Conventional diesel generator
PDR: Price-based DR
UG: utility grid
IRV: Input random variable
$\:{\gamma\:}_{t}\:$: Locational Marginal Prices
$\:{P}_{{UG}_{t}}$: Power transaction with utility grid (kW)
$\:{a}_{i},\:{b}_{i}$: Generation cost coefficients of generator$\it \:\mathrm{i}$
$\:{P}_{{i}_{t}}$: Power generated from the $\it \:{\mathrm{i}}^{\mathrm{t}\mathrm{h}}\:$ generator at time$\it \:\mathrm{t}$
$\:{\theta\:}_{j}$: Customer $\it \:\mathrm{j}$ type between 0 and 1
$\:k_{{1_{j} }} ,k_{{2_{j} }}$: Customer $\it \:\mathrm{j}$ cost coefficients
$$\:{B}_{1,j}\:$$: Customer $$\it \:\mathrm{j}$$ benefit
$\:{{P}_{c}}_{j}$: Customer $\it \:\mathrm{j}$ power curtailment
$\:{B}_{2,j}$: MG operator benefit
$\:{\lambda\:}_{j,t}$: $\it \:{\mathrm{j}}^{\mathrm{t}\mathrm{h}}\:$customer power interruption cost
$\:{P}_{BESS,t}$: Battery storage power
$$\:{P}_{dis,t}$$: Discharging power
$\:{P}_{ch,t}$: Charging power (to the battery)
$$\:{\eta\:}_{dis}$$: Discharging efficiency
$$\:{\eta\:}_{ch}$$: Charging efficiency
$\:TCPD$: BESS total cost per day
$$\:r$$: Interest rate
$$\:LT$$: Battery’s lifetime
$\:{C}_{cap}$: BESS capital cost
$\:{C}_{MC}$: BESS maintenance cost
$$\:ES$$: BESS size
$\:{P}_{W{T}_{t}}$: Hourly wind turbine power
$\:{{P}_{PV}}_{t}$: Hourly PV power
$\:{P}_{{i}_{min}}$and$\:{P}_{{i}_{max}}$: Minimum and maximum generation limits of generator$\it \:\mathrm{i}$
$\:{DR}_{i}\:$and$\:{UR}_{i}$: Ramp down and up rates of generator$\it \:\mathrm{i}$
$\:{P}_{{UG}_{max}}$: Maximum rate of power transaction with the utility grid
$\:{CM}_{j}$: Daily curtailment limit of customer$\it \:\mathrm{j}$
$\:UBL$: Daily budget limit
$\:{SoC}_{min}$and$\:{SoC}_{max}$: BESS minimum and maximum state of charge
$$\:{\xi\:}_{t,k}$$: Standard locations
$$\:{\:p}_{t,k}$$: variable $$\it \:{\mathrm{p}}_{\mathrm{t}}$$ locations
$\:{\mu\:}_{{p}_{t}}$: Mean of output variable$\it \:\:{\mathrm{p}}_{\mathrm{t}}$
$\:{\sigma\:}_{z}$: Standard deviation of variable$\it \:\:{\mathrm{p}}_{\mathrm{t}}$
$$\:\:\:{w}_{t,k}$$: Location weight
$$\:E\left({Y}^{j}\right)$$: Moment vector
